# Albumin-bound paclitaxel drives a cytotoxic CD8^+^ T cell enriched immune microenvironment in triple negative breast cancer

**DOI:** 10.3389/fimmu.2026.1765165

**Published:** 2026-02-18

**Authors:** Daiqin Luo, Xianhuai Jin, Shuai Zhang, Xianlin Zeng, Shuling Zhang, Daohong Li, Wei Xiong, Yan Luo, Zuquan Hu, Jinhua Long, Zhu Zeng

**Affiliations:** 1 School of Biology & Engineering, Guizhou Medical University, Guiyang, China; 2 Department of Oncology, Affiliated Hospital of Guizhou Medical University, Guiyang, China; 3 Department of Oncology, Affiliated Cancer Hospital of Guizhou Medical University, Guiyang, China; 4 Department of Interventional Radiology, Affiliated Hospital of Guizhou Medical University, Guiyang, China; 5 Engineering Center of Cellular Immunotherapy of Guizhou Province, Guiyang, China; 6 Key Laboratory of Microbio and Infectious Disease Prevention & Control of Guizhou, Guiyang, China; 7 Key Laboratory of Infectious Immunity and Antibody Engineering of Guizhou Province, Guiyang, China; 8 Key Laboratory of Endemic and Ethnic Diseases, Ministry of Education, Guizhou Medical University, Guiyang, China; 9 National Key Laboratory for the Exploration and Utilization of Functional Components in Traditional Chinese Medicine, Guizhou Medical University, Guiyang, China; 10 School of Public Health, Guizhou Medical University, Guiyang, China

**Keywords:** dendritic cells, ICD, immune response against cancer, nab-PTX, TNBC

## Abstract

**Background:**

Triple-negative breast cancer (TNBC) is an aggressive subtype of breast cancer characterized by high metastatic potential and resistance to conventional therapies, representing a significant clinical challenge. Although nano albumin-bound paclitaxel (nab-PTX) has demonstrated generally good treatment effect, the mechanisms underlying its enhanced therapeutic performance, particularly its potential immunomodulatory effects, remain unclear.

**Methods:**

Using both *in vitro* and *in vivo* TNBC models, we investigated the immunomodulatory effects of nab-PTX. Specifically, we evaluated its ability to induce immunogenic cell death (ICD), activate dendritic cells (DCs) via the cGAS-STING signaling pathway, and influence CD8^+^ T cell recruitment and infiltration within the tumor microenvironment.

**Results:**

Treatment with nab-PTX induced ICD in TNBC cells was associated with enhanced activation of DCs through the cGAS-STING pathway. This activation was accompanied by improved antigen presentation and a significant increase in intratumoral CD8^+^ T cell infiltration. Collectively, these immune alterations suggest that nab-PTX contributes to a more immunologically active tumor microenvironment, characterized by heightened T cell mediated immune engagement.

**Conclusion:**

Our study indicate that, beyond its direct cytotoxic effects, nab-PTX may exert anti-tumor activity in TNBC through modulation of the tumor immune microenvironment. By inducing ICD and promoting DCs activation, nab-PTX appears to support CD8^+^ T cell recruitment, thereby potentially enhancing immune mediated tumor regression. This immunologically supportive role of nab-PTX highlights its potential value in strategies aimed at improving the efficacy of chemotherapy based or immunotherapy combined treatments in TNBC.

## 1 Introduction

Breast cancer is the second most common malignancy in women, with triple-negative breast cancer (TNBC) comprising 10–20% of cases (1). Defined by the absence of estrogen receptors (ER), progesterone receptors (PR), and human epidermal growth factor receptor 2 (HER2) (2), TNBC is unresponsive to endocrine or HER2-targeted therapies and is characterized by high metastatic potential, chemotherapy resistance, and poor prognosis (3, 4), underscoring the urgent need for improved treatments.

Albumin-bound paclitaxel (nab-PTX), a nanoparticle formulation of paclitaxel bound to human serum albumin, has demonstrated superior efficacy compared with conventional paclitaxel in TNBC (5). Nab-PTX exhibits enhanced pharmacokinetics and tumor penetration and is frequently combined with immune checkpoint inhibitors (6, 7). Although its clinical benefits are well recognized, the molecular basis of its antitumor activity remains incompletely understood. Nab-PTX efficacy may stem from its ability to induce immunogenic cell death (ICD) (8, 9), a regulated process that activates adaptive immunity. Under pathological conditions such as necrosis or cellular stress, dying tumor cells release or expose damage-associated molecular patterns (DAMPs) (10), including surface calreticulin (CRT), extracellular ATP, heat shock protein 70 (HSP70), and nuclear proteins such as high mobility group box 1 (HMGB1). These molecules engage pattern recognition receptors on antigen-presenting cells, particularly dendritic cells (DCs), promoting antigen uptake, processing, and presentation (11, 12). This enhances tumor immunogenicity, remodels the immune microenvironment, and converts “cold” tumors into “hot,” improving immunotherapy response (13, 14).

Given these insights, we hypothesize that nab-PTX augments antitumor immunity in TNBC via ICD induction and DCs activation. To test this, we investigated how nab-PTX-treated TNBC cell-derived products influence DCs function and explored the underlying molecular mechanisms driving this interaction.

## 2 Materials and methods

### 2.1 Cell cultures

Human MDA-MB-231 and mouse 4T1 cell lines were obtained from the Chinese Academy of Sciences (Shanghai, China). The MDA-MB-231 cells were maintained in complete Leibovitz L15 medium (Gibco, USA), while 4T1 cells were cultured in complete Dulbecco’s modified Eagle medium (Gibco, USA). Cells were incubated at 37 °C in a humidified 5% CO_2_ atmosphere.

### 2.2 Isolations and generations of PBMCs and human DCs

PBMCs were isolated from heparinized blood of healthy donors using Ficoll-Paque density gradient centrifugation. To induce immature dendritic cells (imDCs), the harvested PBMCs were cultured in the presence of recombinant human GM-CSF (rhGM-CSF) and interleukin-4 (rhIL-4) over a 5-day differentiation period. Mature dendritic cells (mDCs) were subsequently induced by exposing imDCs to tumor necrosis factor-alpha (TNF-α) and interferon-gamma (IFN-γ).

### 2.3 Isolation and culture of CD3^+^T cells from healthy human peripheral blood

From this same PBMC pool, a portion was differentiated into immature DCs, while CD3^+^ T cells were simultaneously isolated via immunomagnetic separation. This approach ensured that the DCs and T cells used in any given co-culture experiment were autologous. The purified cell pellet was carefully reconstituted in 10 mL of ImmunoCult-XF T Cell Expansion Medium and cultivated under tightly controlled physiological conditions within T75 culture flasks.

### 2.4 Cell viability assay

Cell viabilities under gradient concentrations of nab-PTX (1 nM-100 µM) (YBH16872021, Hengrui Medicine Co., Ltd) exposure were systematically quantified employing the CCK-8 assay kit (Abcam, USA) in strict adherence to the manufacturer’s standardized protocols.

### 2.5 Apoptosis analysis by flow cytometry

The cell apoptosis was quantified through flow cytometry employing an Annexin V-FITC/PI apoptosis detection kit (Merck Millipore, USA) after a 48-hour treatment period with incremental concentrations of nab-PTX.

### 2.6 Transwell assay

DCs were pretreated with conditioned medium for 24 hours before the migration assay to assess their motility. After incubation, DCs were seeded into the upper chambers of 6-well BD Falcon^®^ Transwells. After 24 hours, migrated cells were counted. Migration efficiency was calculated as (migrated DCs/total DCs) ×100%, providing a quantitative measure of motility.

### 2.7 Western blot assay

Western blotting was performed according to standard protocols. Briefly, total protein was extracted after treatment, separated by sodium dodecyl sulfate-polyacrylamide gel electrophoresis, and transferred to nitrocellulose membranes. Membranes were incubated overnight with primary antibodies at specified dilutions, including anti-cyclic GMP-AMP synthase (cGAS) (1:10,000, Abcam, USA, ab302671), anti-stimulator of interferon genes (STING) (1:10,000, Abcam, USA, ab239074), anti-phospho-STING (1:10,000, Cell Signaling Technology, USA, 50907T), anti-TANK-binding kinase 1 (TBK1) (1:10,000, Abcam, USA, ab40676), anti-phospho-TBK1 (1:10,000, Abcam, USA, ab109272), anti-interferon regulatory factor 3 (IRF3) (1:10,000, Abcam, USA, ab68481), and anti-phospho-IRF3 (1:10,000, Abcam, USA, ab76493). Protein band intensities were quantified using Image J for statistical analysis.

### 2.8 Immunohistochemistry

Tumor specimens were embedded in paraffin blocks and incubated with anti-CD11c primary antibody (diluted at 1:100 ratio; CST #97586S, Cell Signaling Technology, USA) at 4 °C for 16 hours. Specimens were then treated with HRP-conjugated secondary antibody and imaged using high-resolution digital microscopy.

### 2.9 Immunofluorescence

Cells were immunostained with primary antibodies: anti-HMGB1 (1:100 dilution, Abcam, USA, ab18256) and anti-CD11c (1:100 dilution, Abcam, USA, ab52632). Samples were then treated with Alexa Fluor 488-conjugated secondary antibody (1:1000 dilution, Thermo Fisher Scientific, USA) followed by nuclear counterstaining with 4’,6-diamidino-2-phenylindole (DAPI).

### 2.10 Total RNA extraction and quantitative real-time PCR

Total RNA was extracted from cellular specimens utilizing TRIzol™ Reagent (Thermo Fisher Scientific, USA) in strict accordance with the manufacturer’s guidelines. Subsequently, first-strand cDNA synthesis was performed with the High Capacity cDNA Reverse Transcription Kit (Thermo Fisher Scientific, USA). Quantitative reverse transcription polymerase chain reaction (qRT-PCR) assays were carried out on a Bio-Rad CFX platform using iTaq Universal SYBR Green (Bio-Rad, USA), with primer sequences specified in **Table 1**.

**Table 1:** Primers used in this study.

Primer ID	Sequence (5’-3’)
HMGB-1-F	AAATGAAAACCTATATCCCTCCC
HMGB-1-R	GGGCGATACTCAGAGCAGAAG
CRT –F	AGATAAAGGTTTGCAGACAAGC
CRT –R	CATGTCTGTCTGGTCCAAACTA
Hsp70A1A –F	GACTCCCGTTGTCCCAAG
Hsp70A1A -R	CGGTTCCCTGCTCTCTGT
Caspase-3-F	GGAACAAATGGACCTGTTGAC
Caspase-3-R	CTCAATGCCACAGTCCAGTTC
CD80-F	GTGGTCACAATGTTTCTGTTGA
CD80-R	GTTCTTGTACTCGGGCCATATA
CD86-F	TGCTCATCTATACACGGTTACC
CD86-R	TGCATAACACCATCATACTCGA
CD40-F	TCACCTCGCTATGGTTCGTC
CD40-R	GGAAGGCATTCCGTTTCAGT
HLA-DR –F	CCAGAGACTACAGAGAATGTGG
HLA-DR –R	TTGATGATGAAGATGGTCCCAA
GAPDH –F	GACCTGACCTGCCGTCTA
GAPDH –R	AGGAGTGGGTGTCGCTGT

### 2.11 Enzyme-linked immunosorbent assay

The analytical measurements were performed using commercially available ELISA kits, with all protocols meticulously followed according to manufacturers’ specifications. Optical density values were then precisely measured through microplate spectrophotometry at designated wavelengths.

### 2.12 Xenograft models

Female BALB/c mice (6–8 weeks old, 17 ± 3g body weight) sourced from Animal Experimental Center of Guizhou Medical University, were housed in room temperature (20-26 °C) and specific pathogen-free (SPF) environment. To avoid rejection and foreign body response, BALB/c mice underwent subcutaneous implantation of mouse 4T1 cells in the right flank region. When tumors reached an average volume of ~100 mm^3^, the mice were randomly assigned to two experimental cohorts: a control group receiving PBS solution and a treatment group administered nab-PTX via therapeutic intraperitoneal injection on days 7, 11, and 15. After 19 days post-tumor injection, the mice were euthanized by isoflurane inhalation (Isofluorane, R510-22, RWD Life Science Co, China), and the tumors were removed and weighed.

### 2.13 Co-culture of imDCs with conditioned medium

Conditioned media from MDA-MB-231 cells, treated with 5 nM nab-PTX for 48 hours or untreated controls (NC) in Leibovitz L15 medium, were added to 6-well plates. ImDCs were cultured in these conditioned media (imDCs+nab-PTX and imDCs+NC) under standard conditions. After 48 hours, cells were harvested for further experiments.

### 2.14 Analyses of T cell subsets

DCs were primed with conditioned medium and co-cultured with T cells at a 1:10 ratio for 48 hours. After incubation, supernatants were collected and aliquoted into sterile microcentrifuge tubes for ELISA.

### 2.15 Bioinformatics analysis

Proteomic data were collected from tumor tissues of three mice per group (nab-PTX treated and control) and analyzed by liquid chromatography-mass spectrometry (LC-MS). Differentially expressed proteins were identified through comparative profiling and subsequently subjected to Gene Ontology (GO) classification and Kyoto Encyclopedia of Genes and Genomes (KEGG) pathway enrichment analyses. Functional annotation was performed using EggNOG-mapper (v2.0) for GO categorization, and KEGG pathway annotation was conducted via BLAST searches against the KEGG database. The disease-free survival (DFS) and overall survival (OS) were evaluated by the Gene Expression Profiling Interactive Analysis 2 (GEPIA 2) platform (http://gepia2.cancer-pku.cn/#survival). Prognostic correlations across subtypes, with a specific emphasis on TNBC, were examined using bc-GenExMiner v4.9. Protein-protein interaction networks were mapped via the STRING database (https://cn.string-db.org/), while UALCAN (https://ualcan.path.uab.edu/) facilitated comparative analyses of GZMB and CD8α expression profiles between malignant and adjacent normal tissues.

### 2.16 Statistical analysis

All the data were analyzed from at least three independent experiments and the quantification results were expressed as mean ± SD. Data met the assumption of the statistic tests. Statistical comparisons between two groups were conducted by using the Student’s *t* test and between multiple groups using one or two-way ANOVA using the Prism software (v.7.0, GraphPad, San Diego, CA), whereas image J program was used for picture analysis. Statistical significance was defined as *p < 0.05.

### 2.17 nab-PTX induced ICD of TNBC *in vitro*

**Figure 1:**
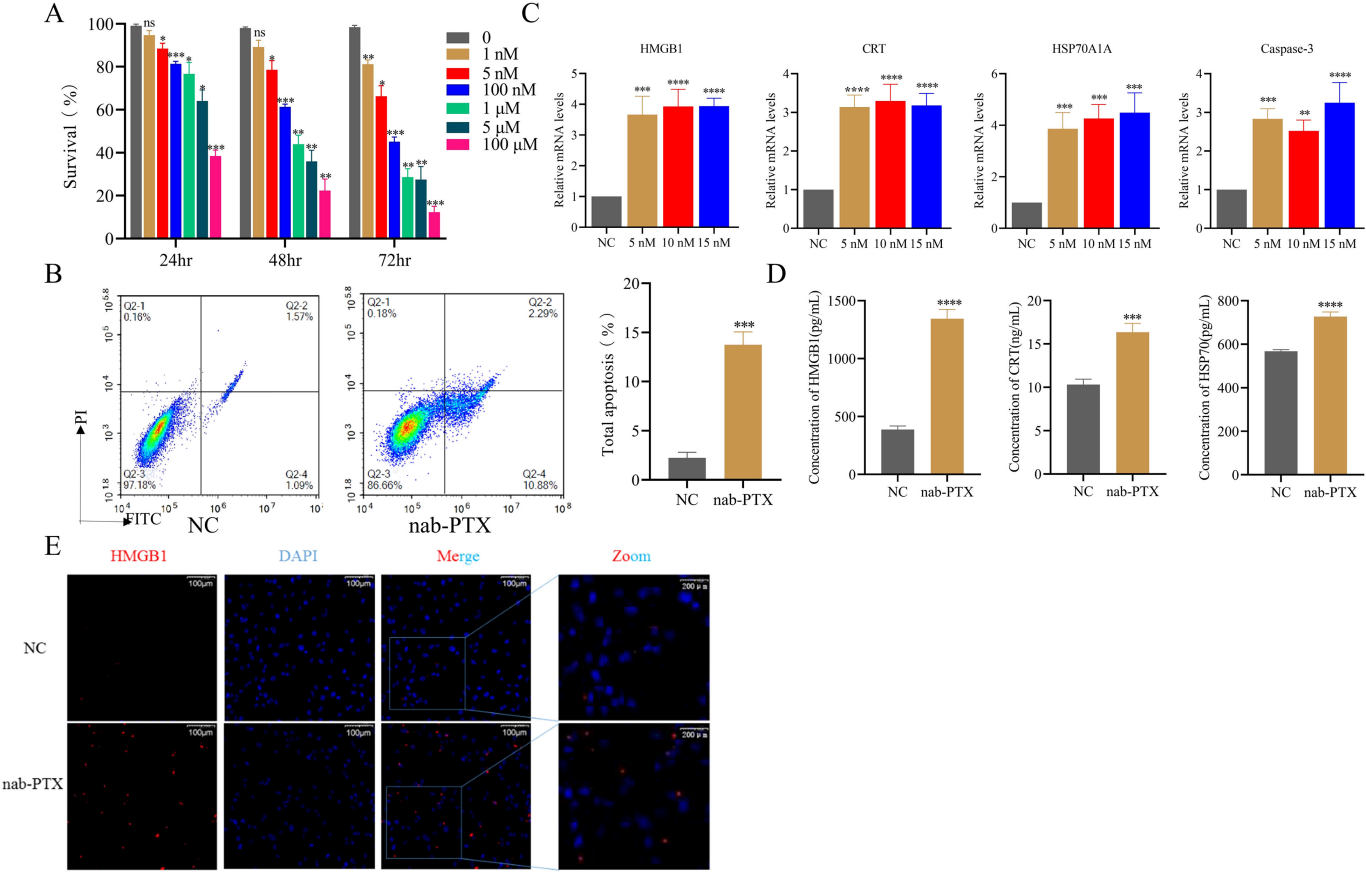
Effects of albumin-paclitaxel on the immunogenic cell death of MDA-MB-231 cells. **(A)** Cell viability of MDA-MB-231 cells treated with varying concentrations of nab-PTX was assessed at 24, 48, and 72 hours. **(B)** Apoptosis was evaluated by Annexin V/PI staining, with representative flow cytometry plots and quantification shown after 24 h of 5 nM nab-PTX treatment. **(C)** Expression of ICD-related genes, including HMGB1, CRT, HSP70A1A, and Caspase-3, was analyzed by RT-qPCR. **(D)** ELISA measurement of ICD-related proteins in MDA-MB-231 culture medium. **(E)** Visualization of HMGB1 as detected by anti-HMGB1 immunofluorescence (red). In **(C-E)**-related experiments, MDA-MB-231 cells were treated with nab-PTX (5 nM) for 24 h. * *p*<0.05, ** *p*<0.01, *** *p*<0.001 , **** *p*<0.0001, versus control. ns, not significant.

To determine the optimal nab-PTX treatment conditions, MDA-MB-231 cells were exposed to graded concentrations. CCK-8 assays revealed a significant, dose- and time-dependent inhibition of cell proliferation (**Figure 1A**, P < 0.001). Treatment with 1 nM for 24, 48, and 72 hours resulted in proliferation rates of approximately 80-90%. When the concentration was increased to 5 nM for the same durations, proliferation decreased to roughly 60-80%. Treatment with 100 nM or higher concentrations led to a more pronounced reduction in proliferation; notably, after exposure to 100 μM for 72 hours, the cells were almost completely unable to adhere, and the proliferation rate dropped to around 10%. Based on these findings, subsequent experiments were conducted using untreated controls and cells treated with 5 nM nab-PTX for 48 hours. Flow cytometry analysis confirmed that nab-PTX induced apoptosis (**Figure 1B**). To further evaluate its immunogenic potential, RT-qPCR was performed to quantify canonical ICD biomarkers (HMGB1, CRT, HSP70A1A, and Caspase-3), which were significantly upregulated across treatment groups (**Figure 1C**). Consistently, ELISA demonstrated elevated extracellular levels of HMGB1, CRT, and HSP70 in the supernatants of nab-PTX–treated cells (**Figure 1D**). Immunofluorescence imaging additionally revealed nuclear-to-cytoplasmic HMGB1 translocation in treated cells compared with controls (**Figure 1E**). Collectively, these findings demonstrate that nab-PTX triggers the release of ICD associated DAMPs.

### 2.18 The conditioned medium stimulated the maturation of imDCs and the cytokine secretion of DCs

**Figure 2:**
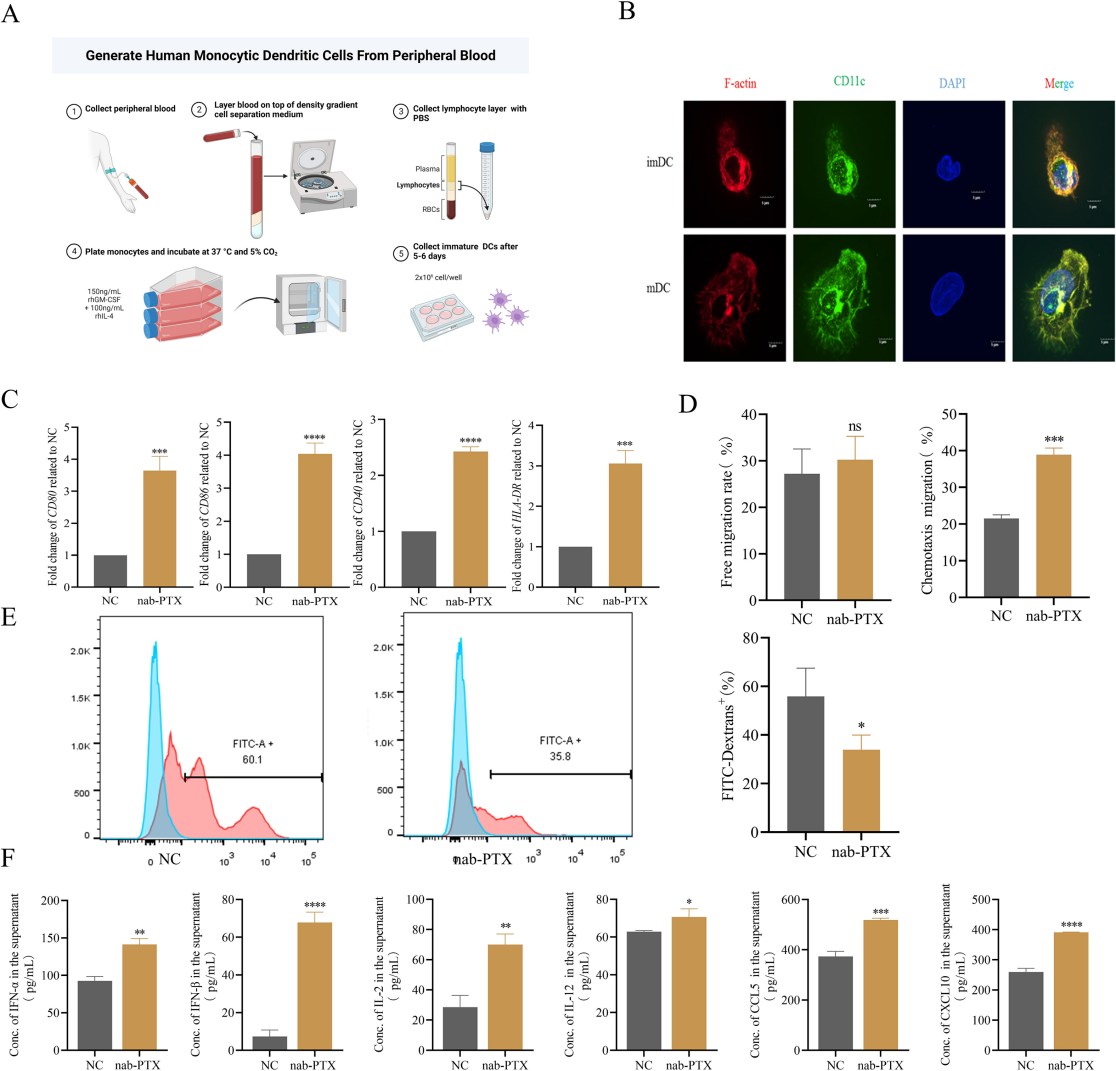
The conditioned medium could induce the maturation of imDCs and stimulate DCs to secrete cytokines. **(A)** imDCs were isolated from peripheral blood of healthy donors. **(B)** Immature and mature DCs were analyzed by confocal microscopy. **(C-F)** imDCs were incubated for 24 h in medium alone (NC) or in the conditioned medium (nab-PTX) (n=3 in each group). **(C)** CD80, CD86, CD40, and HLA-DR expression in DCs was determined by real-time PCR. **(D)** The Transwell migration assay was used to detect the free migration ability and the chemotactic ability towards 250ng/mL CCL19 of DCs *in vitro*. **(E)** Flow cytometry analysis of DCs phagocytosis of FITC-labeled dextrans. **(F)** Cytokine levels such as IFN-α,IFN-β,IL-2,IL-12,CCL-5,and CXCL10 in supernatants from DCs cultures measured by ELISA. * *p*<0.05, ** *p*<0.01, *** *p*<0.001 , **** *p*<0.0001, versus control. ns, not significant.

Given the central role of DCs in T cell activation, we investigated the impact of conditioned medium on DC maturation. PBMCs from healthy donors were differentiated into imDCs and mDCs using standard protocols (**Figure 2A**). Confocal microscopy confirmed cell integrity and distinct morphology (**Figure 2B**). Conditioned medium from MDA-MB-231 cells exposed to nab-PTX for 48 hours was used in subsequent experiments. ImDCs cultured in conditioned medium showed significant upregulation of maturation markers (CD80, CD86, CD40, HLA-DR) compared with controls, as measured by qRT-PCR (**Figure 2C**). Transwell assays compared migratory behavior under different conditions. Spontaneous migration was similar between controls (23-33%) and treated groups (24-35%), but conditioned medium–treated DCs exhibited enhanced chemotaxis (**Figure 2D**). Phagocytosis assays revealed reduced FITC-dextran uptake in conditioned medium-treated DCs (**Figure 2E**). Cytokine profiling showed increased secretion of IFN-α, IFN-β, IL-2, IL-12, CCL5, and CXCL10 in treated groups (**Figure 2F**). Collectively, these findings demonstrate that conditioned medium promotes imDC maturation into mDCs, characterized by enhanced chemotaxis and elevated cytokine and chemokine secretion.

### 2.19 ImDCs that exposed in conditioned medium stimulated the cytokines secretion of CD8^+^T cells

**Figure 3:**
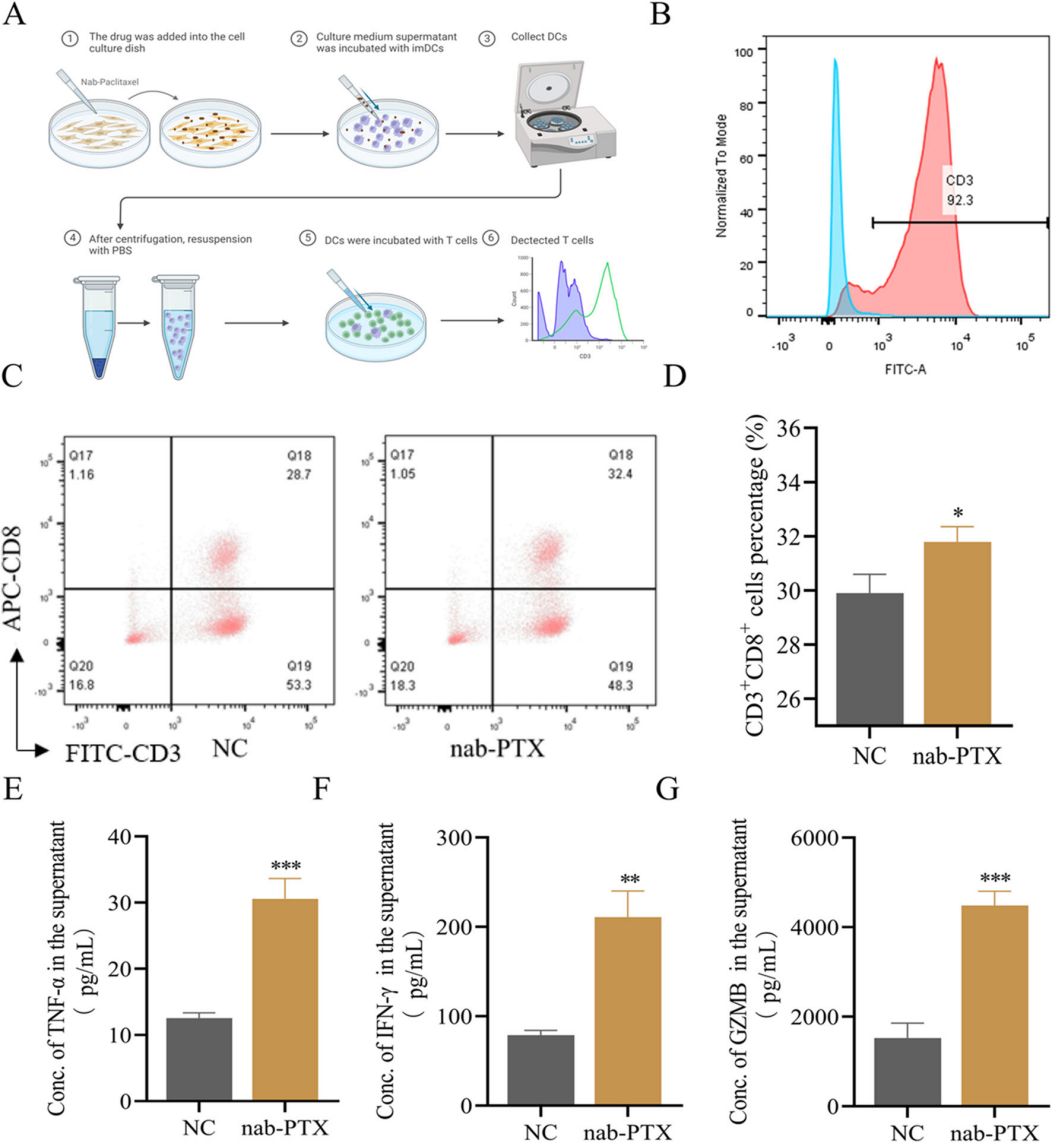
Effects of DCs stimulated by conditioned medium on T cells. **(A, B)** Human CD3^+^ T cells sorting and identification. **(A)** Flow chart of classification of T cells after co-incubation of DCs and T cells stimulated by conditioned medium. **(B)** Human CD3^+^ T cells were isolated by magnetic beads and identified by FCM for purity, their purity was>90%. **(C, D)** Flow cytometry was used to detect the differentiation of T cells after co-incubation of DCs which stimulated by conditioned medium. **(C)** Flow cytometry images; **(D)** Statistical figure graphed using GraphPad Prism 6.0. **(E-G)** DCs stimulated by conditioned medium stimulated T cells to secrete TNF-α, IFN-γ and GZMB. *p < 0.05, **p < 0.01, ***p < 0.001 versus control.

Conditioned medium-activated imDCs were co-cultured with purified T cells for 24 hours (**Figure 3A**). To evaluate the immunomodulatory effects on T cell proliferation, CD3^+^T cells were isolated from PBMCs using magnetic sorting, reaching 92.3% purity confirmed by flow cytometry (**Figure 3B**). Co-culture with stimulated DCs significantly increased CD3^+^CD8^+^T cell subsets, rising from 28.7% in controls to 32.4% in the experimental group (**Figure 3C, D**) (*p*<0.05). These results suggest that conditioned medium–activated imDCs promote T cell differentiation. To assess cytokine secretion, TNF-α, IFN-γ, and Granzyme B (GZMB) were quantified by ELISA. TNF-α secretion increased from 12.1 ± 1.3 pg/mL in controls to 30.4 ± 2.7 pg/mL in treated groups (**Figure 3E**). IFN-γ levels rose from 79.8 ± 6.5 pg/mL to 198.2 ± 12.1 pg/mL (**Figure 3F**). GZMB production increased 2.1-fold in treated groups compared with controls ((**Figure 3G**). Collectively, these findings show that conditioned medium–activated imDCs enhance T cell effector responses by upregulating pro-inflammatory cytokines and cytotoxic mediators.

### 2.20 nab-PTX induced more infiltrations of DCs and CD8^+^ T cells in the cancer tissue of TNBC mouse model

**Figure 4:**
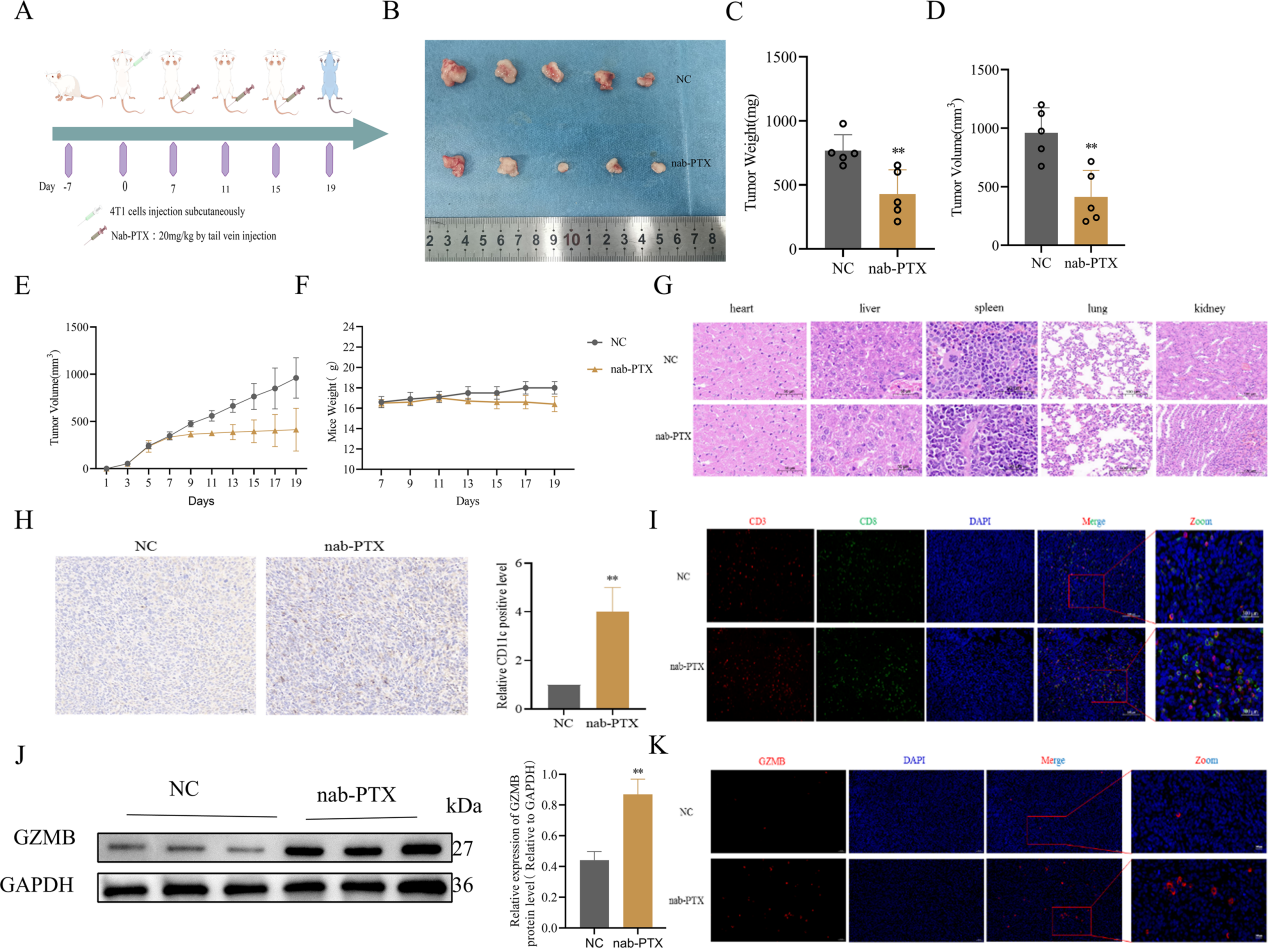
nab-PTX promotes CD8^+^T cell infiltration in 4T1 Balb/c mice model tumors *in vivo*. **(A)** Schematic design of mice TNBC model. Balb/c mice were acclimated to housing environment for 7 days followed by a injection of 5×10^5^ 4T1 cells under the mammary fat pad. The mice were then randomly sub-grouped for systemic treatments by nab-PTX (20 mg/kg), and the control group was infused with saline for 19 days. **(B)** Tumor size, **(C)** weight, **(D)** and volume in Balb/c tumor-bearing mice. **(E)** Tumor volume curve of Balb/c tumor-bearing mice. **(F)** Body weight growth curve of Balb/c tumor-bearing mice. **(G)** HE-stained tissue specimens of heart, liver, spleen, lung, and kidney from mice showing no toxic damage. **(H)** Immunohistochemistry for the expression of the DC marker CD11c in Balb/c mice tumors (n=5). **(I)** The representative immunofluorescence image of CD3^+^ and CD8^+^ T cell infiltration in Balb/c mice tumor tissues. **(J)** Assessment of the effect of the indicated treatment regimens on GZMB in the Balb/c mice tumors. Representative western blot images and quantification of GZMB. **(K)** The representative immunofluorescence image of GZMB infiltration in Balb/c mice tumor tissues. **p < 0.01, versus Control.

To assess the immunomodulatory effects of nab-PTX *in vivo*, TNBC mouse models were generated by orthotopic implantation of 4T1 cells into BALB/c mammary fat pads, with a schematic summarizing dosing and sample collection (**Figure 4A**). During the 19-day treatment, longitudinal monitoring showed comparable physiological profiles, particularly body weight, between nab-PTX and saline groups (**Figure 4F**). Quantitative analysis showed significantly greater tumor suppression in nab-PTX-treated mice, with marked reductions in tumor volume (**Figures 4B, D, E**) and mass (**Figure 4C**) compared with controls. HE staining confirmed no toxic damage in major organs, including heart, liver, spleen, lung, and kidney (**Figure 4G**). Immunohistochemistry revealed robust DC infiltration in nab-PTX-treated tumors, with a 2.8-fold increase in CD11c^+^cell density compared with controls (**Figure 4H**). Immunofluorescence showed increased tumor-infiltrating CD8^+^T cells after nab-PTX treatment (**Figure 4I**). Molecular profiling revealed strong upregulation of GZMB in treated tumors, with western blot confirming a 2.1-fold protein increase versus controls (*p* < 0.01, **Figures 4J, K**). These findings demonstrate that nab-PTX enhances antitumor immunity by promoting DC activation and cytotoxic T cell-mediated tumor elimination.

### 2.21 CD8ɑ and GZMB expressions are positively correlated with DFS and OS in breast cancer and TNBC subtype

**Figure 5:**
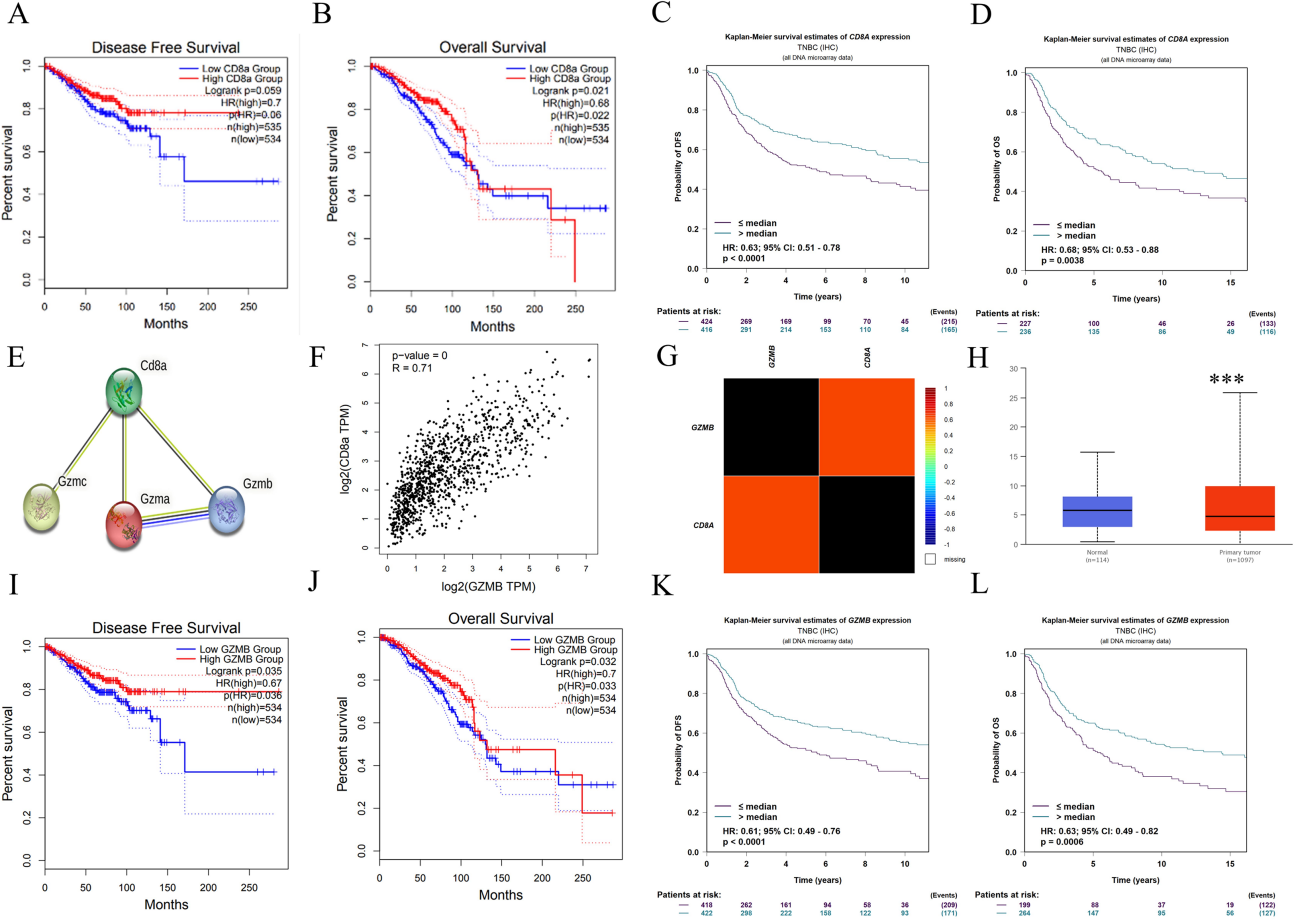
Survival and prognostic values of CD8a and GZMB. Kaplan-Meier curve estimates of the DFS **(A)** and OS **(B)** of breast cancer patients at different CD8a gene levels; Kaplan-Meier curve estimates of the DFS **(C)** and OS **(D)** of TNBC patients at different CD8a gene levels; **(E)** CD8a protein interacts with granzyme protein family; **(F-G)** CD8a gene was positively correlated with GZMB gene; **(H)** CD8a gene is low expressed in breast cancer; Kaplan-Meier curve estimates of the DFS **(I)** and OS **(J)** of breast cancer patients at different GZMB gene levels; Kaplan-Meier curve estimates of the DFS **(K)** and OS **(L)** of TNBC patients at different CD8a gene levels. ***p < 0.001, versus control.

To identify key prognostic factors in TNBC, the expression levels of CD8ɑ gene and its clinical implications were investigated. The data revealed that elevated CD8ɑ expression correlated with improved DFS and OS (**Figure 5A, B**). Statistical analysis confirmed significantly enhanced DFS and OS in the high CD8ɑ expression group compared to the low-expression cohort (**Figure 5C, D**). Notably, CD8ɑ downregulation was consistently observed in breast cancer tissues versus adjacent normal tissues (**Figure 5H**). Given GZMB’s established role as a key cytotoxic effector in CD8^+^ T cell immunity, a strong positive correlation between CD8ɑ and GZMB expression patterns (**Figure 5E-G**) was identified. Survival analysis further demonstrated that GZMB levels emerged as independent predictors of prolonged DFS (*p* = 0.035, HR = 0.67) and OS (*p* = 0.032, HR = 0.7) (**Figure 5I-J**), with this prognostic relationship proving most pronounced in TNBC subtypes (**Figure 5K-L**).

### 2.22 nab-PTX remodeled the cancer immune microenvironment

**Figure 6:**
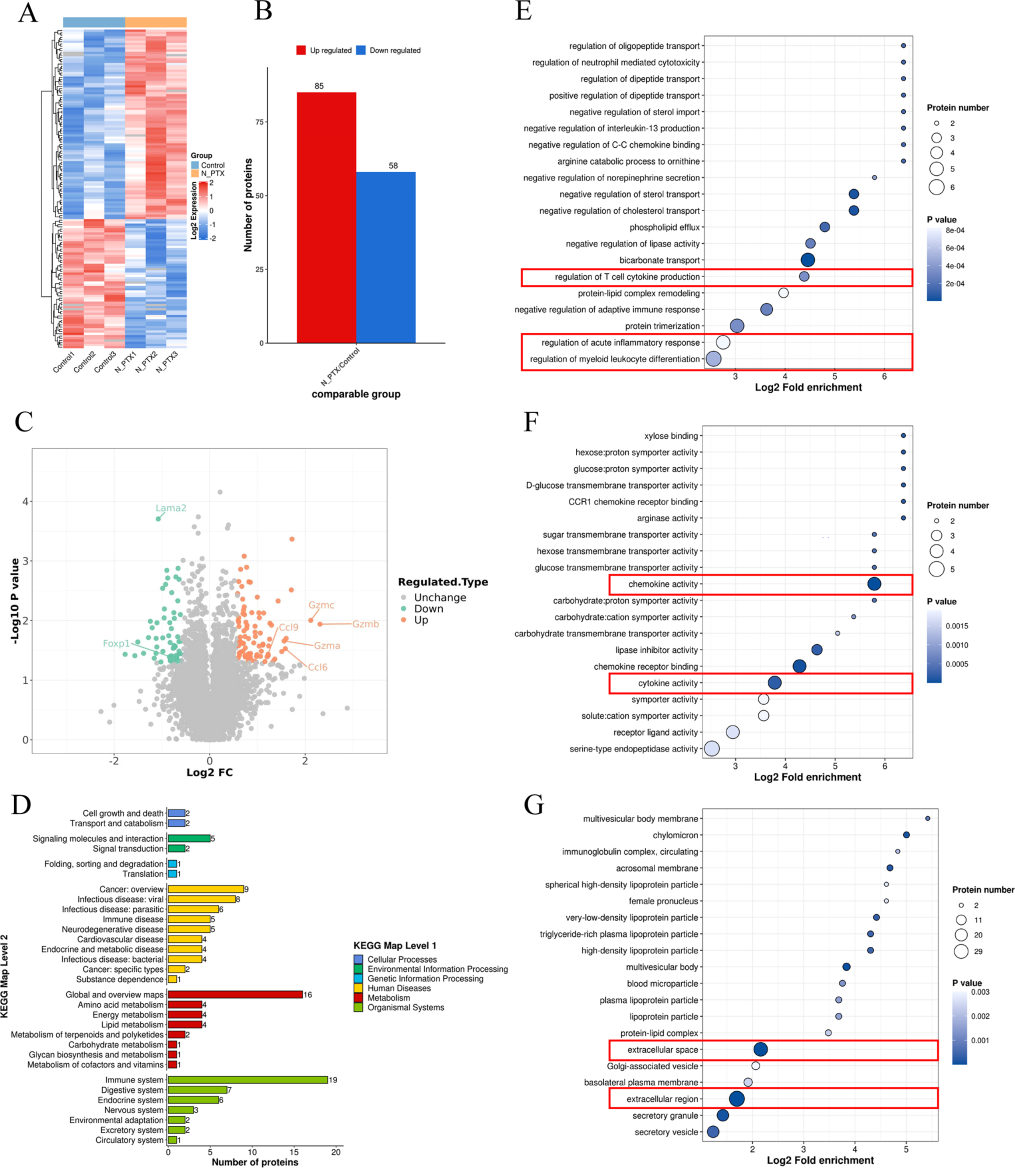
Proteomic map of Balb/c mice tumor tissues. **(A)** Heat map of differentially expressed proteins (DEPs). **(B)** Statistical map of differential expression proteins. **(C)** Differential protein volcano map. **(D)** The biological functions influenced by the differentially expressed proteins. **(E)** DEPs were mainly enriched in inflammatory response, **(F)** chemokine activity, and cytokine activity. **(G)** Subcellular location analysis indicated that DEPs originated from all major cellular components, mainly the extracellular region.

To assess the immunomodulatory effects of nab-PTX *in vivo*, proteomic profiling was performed on tumor tissues from mouse models. Differential expression analysis revealed distinct molecular signatures between groups, illustrated in the hierarchical clustering heatmap (**Figure 6A**). Mass spectrometry identified 7,143 proteins, of which 85 were upregulated and 58 downregulated (**Figure 6B**). Granzyme family members and chemokine-related proteins were among the most strongly upregulated (**Figure 6C**). KEGG pathway analysis showed that upregulated proteins were mainly involved in immune processes (**Figure 6D**). Functional annotation identified three main effects: regulation of bone marrow leukocyte differentiation, acute inflammatory responses, and T-cell cytokine production (**Figure 6E**). Molecular analysis highlighted chemokine activity and cytokine receptor binding as the most affected functions (**Figure 6F**). Subcellular localization showed that 64.3% of differentially expressed proteins were extracellular (**Figure 6G**), suggesting nab-PTX may enhance immune infiltration and antitumor responses via extracellular matrix remodeling.

### 2.23 ImDCs were matured by conditioned medium via cGAS-STING signaling pathway

**Figure 7:**
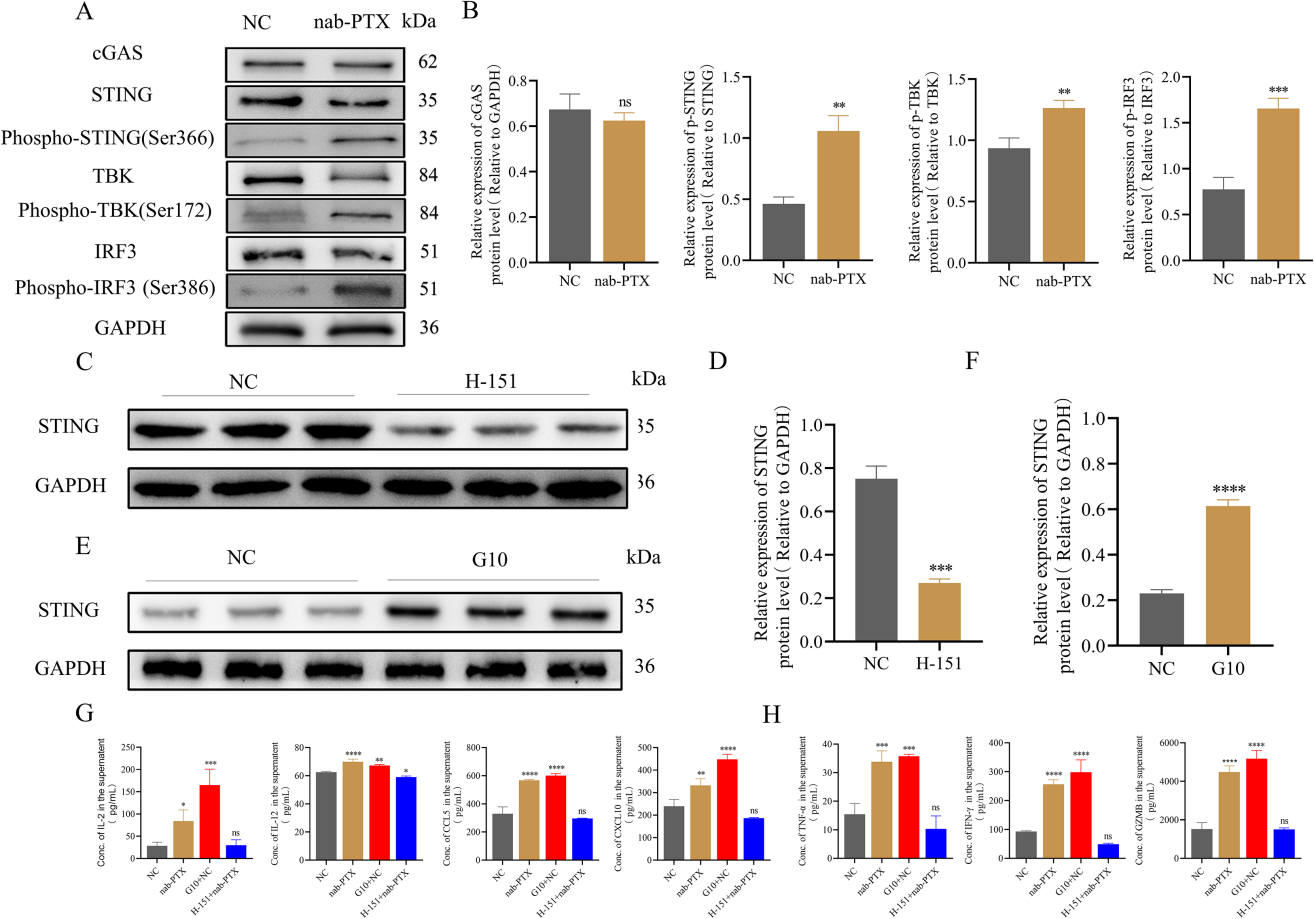
The cGAS-STING signaling pathway of imDCs was activated by conditioned medium to stimulate imDCs into mDCs. **(A, B)** The protein level of the cGAS-STING pathway by Western blot in two groups. **(A)** WB protein expression bands. **(B)** Quantification of WB signals measured as grey values using Image **(J) (C-F)** The expression of STING protein was detected by Western blotting. **(C)** Premedication with a STING protein inhibitor H-151. **(D)** Relative gray scale of WB analysis results in **(C)**. **(E)** Premedication with a STING protein agonist G10. **(F)** Relative gray scale of WB analysis results in **(E)**. **(G)** Cytokine levels such as IL-2, IL-12, CCL5, and CXCL10 in supernatants from DCs cultures measured by ELISA. **(H)** Cytokine levels such as TNF-α and IFN-γ, and the protein level GZMB in supernatants from T cells cultures measured by ELISA. * *p*<0.05, ** *p*<0.01, *** *p*<0.001 , **** *p*<0.0001, versus control. ns, not significant.

Western blot was performed to assess key protein expression in the cGAS-STING pathway of DCs after conditioned media exposure. Quantitative analysis showed marked upregulation of p-STING, p-TBK, and p-IRF3 in conditioned media-treated DCs compared with controls (**Figures 7A, B**). Treatment with H-151, a selective STING inhibitor, reduced STING expression in imDCs (**Figures 7C, D**). Conversely, the STING agonist G10 increased STING expression in complementary experiments (**Figures 7E, F**). Cytokine profiling showed that G10-stimulated imDCs had increased IL-2 and IL-12 secretion, along with elevated CCL5 and CXCL10. In contrast, H-151-treated imDCs maintained baseline IL-2, CCL5, and CXCL10 but showed reduced IL-12 production (**Figure 7G**). The cGAS-STING pathway significantly regulated TNF-α, IFN-γ, and GZMB secretion (**Figure 7H**).

**Figure 8:**
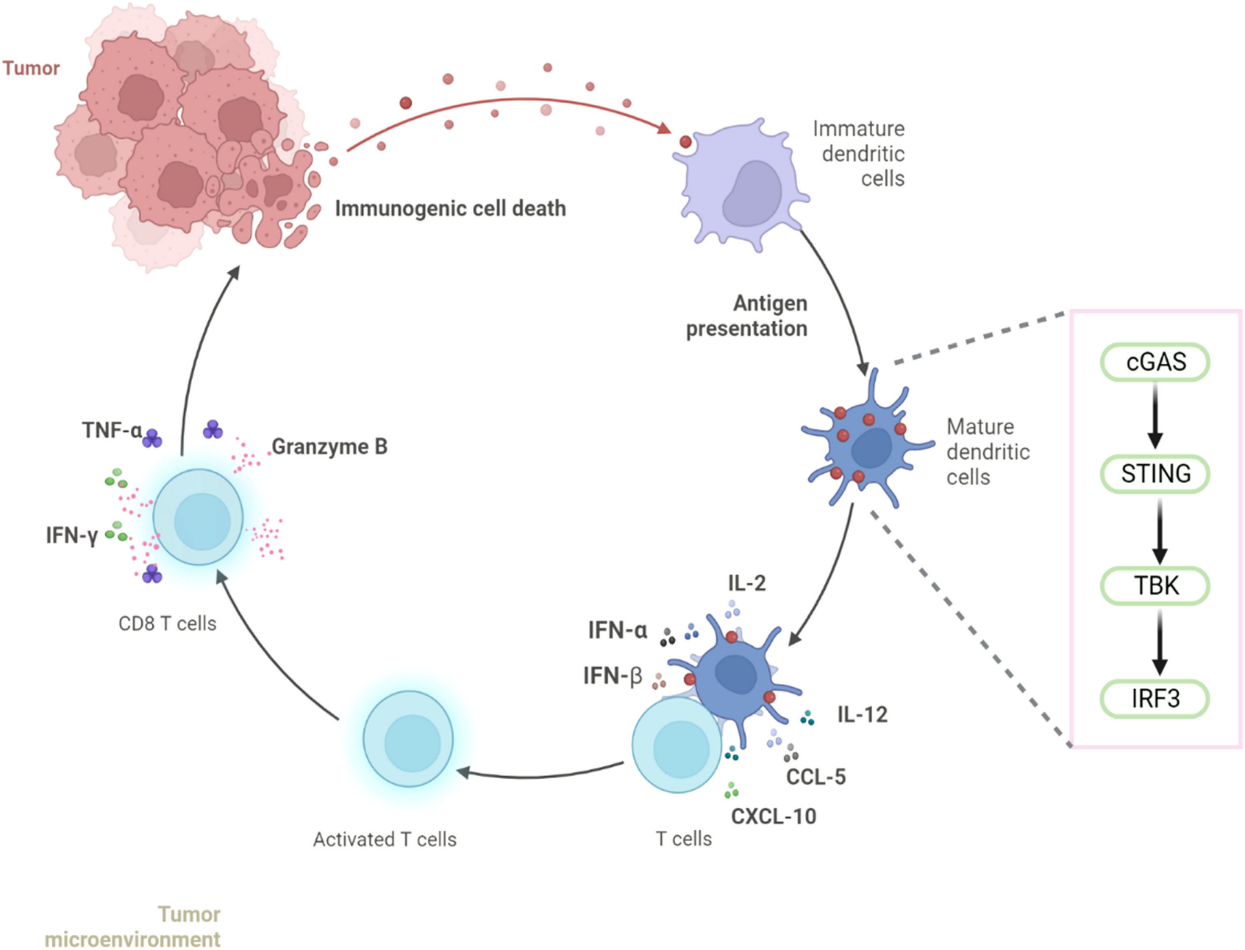
nab-PTX activates the cGAS-STING signaling pathway in imDCs and induces CD8^+^ T-cell infiltration in TNBC.

These findings found that conditioned media from nab-PTX-treated MDA-MB-231 cells enhanced imDC immunogenicity via cGAS-STING activation, promoting maturation and inducing IL-2, IL-12, CCL5, and CXCL10 production. This cytokine response drives CD8^+^T cell differentiation and activation, resulting in synergistic enhancement of anti-tumor immunity (**Figure 8**).

## 3 Discussion

Combining immune checkpoint inhibitors with chemotherapy has emerged as a transformative strategy in oncology, particularly for cancers without targeted therapies, such as TNBC (15). Supported by the 2020 ESMO guidelines (16) and clinical evidence, notably the IMpassion130 study (17), atezolizumab plus nab-PTX is now the first line immunotherapy for PD-L1 positive metastatic TNBC. However, the molecular mechanisms underlying this synergistic clinical efficacy remain incompletely understood. Our study delineates a coherent immunostimulatory pathway activated by nab-PTX in TNBC models. It is important to note that the unique efficacy of nab-PTX in combination with immune checkpoint inhibitors, as evidenced by the success of the IMpassion130 regimen contrasted with the negative outcome of IMpassion131, suggests that its immunomodulatory effects may be quantitatively or qualitatively distinct. Recent translational research has begun to elucidate this difference. Several studies (18–21) compared the tumor immune microenvironment in patients treated with taxanes different combinations. The albumin nanoparticle carrier likely alters drug distribution, enhances tumor penetration, and may facilitate a more potent or sustained immune activation, potentially explaining its status as the preferred partner for chemoimmunotherapy in TNBC.

The immunological basis of chemo-immunotherapy is chemotherapy induced ICD, driven by DAMPs such as CRT, extracellular ATP, and HMGB1 release (22, 23). Preclinical studies support the immunogenicity of taxanes, for example, Lau et al. (24) demonstrated paclitaxel-induced ICD in ovarian carcinoma, while Scribano et al. (25) showed that ~10 nM paclitaxel triggered ICD through chromosomal segregation errors and multipolar spindle formation. Mechanistically, HMGB1 translocates from the nucleus to the cytoplasm during apoptosis, and its extracellular levels positively correlate with ICD progression (26). Consistently, Savage et al. (27) showed that radiotherapy enhances tumor immunogenicity by upregulating CRT and HMGB1, findings supported by studies linking HMGB1 to CD8^+^ T cell function and its overexpression in cancers. In line with this, our data revealed strong extracellular accumulation of HMGB1, CRT, and HSP70 after nab-PTX treatment, indicating that ICD induction likely contributes to its clinical efficacy. In this study, we demonstrate that nab-PTX induces the release of key DAMPs associated with ICD and is functionally linked to the activation of an antitumor immune response. It is important to note that while the observed DAMP profile is highly indicative of ICD, definitive functional proof, remains to be established in future studies.

Intratumoral DCs internalize tumor DNA, activating cGAS-STING signaling to enhance antigen presentation and T-cell activation (28, 29). Evidence identifies this axis as a central mediator of antitumor immunity (30). In our study, DCs exposed to medium from nab-PTX-treated TNBC cells showed strong upregulation of cGAS-STING components with elevated cytokine production. Co-culture with medium from STING-activated DCs increased IL-2, IL-12, CCL5, and CXCL10, whereas medium from STING-inhibited DCs reduced their secretion. This bidirectional effect confirms that conditioned medium acts as a STING agonist. Accordingly, medium from nab-PTX-treated MDA-MB-231 cells activated STING signaling in imDCs, enhancing cytokine secretion and CD8^+^ T-cell cytotoxicity. While our data establish that conditioned medium from nab-PTX treated TNBC cells activates the cGAS-STING pathway in DCs, the specific identity of the key DAMPs responsible for this activation warrants further investigation. Although the release of nuclear DNA is a well characterized consequence of ICD and a canonical ligand for cGAS, future studies employing DNase treatment of conditioned medium or direct measurement of cytosolic DNA accumulation in DCs are needed to conclusively verify DNA as the principal trigger in this context, and to delineate its role relative to other co-released DAMPs.

Most TNBC patients exhibit low tumor-infiltrating lymphocytes, creating a “cold tumor” phenotype with poor therapeutic response (31). Our findings demonstrate that nab-PTX induces CD8^+^T-cell infiltration and reshapes the tumor immune microenvironment in TNBC mice. Beyond its role as a microtubule-stabilizing cytotoxic agent, nab-PTX modulates antitumor immunity, likely by regulating cytokine and chemokine expression. While our *in vitro* data clearly demonstrate nab-PTX conditioned medium activates the cGAS-STING pathway in human DCs, and our *in vivo* proteomic reveal a CD8^+^ T cell inflamed tumor microenvironment consistent with this mechanism, future studies could directly assess the phosphorylation status of STING and IRF3 within tumor infiltrating immune cells from treated animals to provide additional confirmation of the pathway’s engagement in the *in vivo* setting. It is important to note that while our data demonstrate activation of the cGAS-STING pathway in DCs exposed to conditioned medium from nab-PTX treated cells, the strict dependency of the subsequent T cell priming effects on this specific pathway was not genetically or pharmacologically validated in our main co-culture or *in vivo* models. Future studies utilizing cGAS-STING knockout DCs or specific pathway inhibitors in these functional assays will be crucial to establish a definitive causal link. Our conclusions are based primarily on murine models, and their applicability to clinical settings remains uncertain, given immunological and microenvironmental differences between mice and humans. Future work should therefore focus on validating the immunomodulatory effects of nab-PTX in patient samples and elucidating the molecular pathways through which it remodels the tumor immune microenvironment to inform optimized combination immunotherapies.

## 4 Conclusions

In summary, this study demonstrates that nab-PTX induces immunogenic cell death in MDA-MB-231 cells, leading to the release of DAMPs that engage the cGAS-STING pathway in DCs. Activation of this innate immune signaling axis enhances antigen presentation and is associated with increased infiltration of CD8^+^ T cells within the tumor microenvironment, highlighting a functional link between innate immune sensing and adaptive T cell responses. From a tumor immunity perspective, these findings suggest that nab-PTX may contribute to shaping an immune microenvironment that supports T cell mediated antitumor activity rather than acting solely as a cytotoxic agent. Such immune modulation provides a mechanistic basis for combining nab-PTX with T cell directed immunotherapies, including immune checkpoint inhibitors, to potentially enhance antitumor immune responses in TNBC. However, given that the present conclusions are derived from murine models, further validation in human tumors is required. Future studies should focus on integrating clinical samples and combination treatment strategies to delineate how nab-PTX driven innate immune activation can be harnessed to optimize T cell dependent tumor immunity.

## Data Availability

The raw data supporting the conclusions of this article will be made available by the authors, without undue reservation.
